# Caffeine Inhibits Acetylcholinesterase, But Not Butyrylcholinesterase

**DOI:** 10.3390/ijms14059873

**Published:** 2013-05-08

**Authors:** Miroslav Pohanka, Petr Dobes

**Affiliations:** 1 Faculty of Military Health Sciences, University of Defense, Trebesska 1575, 50001 Hradec Kralove, Czech Republic; 2 Regional Centre for Applied Molecular Oncology, Masaryk Memorial Cancer Institute, Zluty Kopec 7, 65653 Brno, Czech Republic; E-Mail: pietroli@seznam.cz

**Keywords:** acetylcholinesterase, butyrylcholinesterase, caffeine, Alzheimer disease, myasthenia gravis, acetylcholine, inhibition, coffee, alkaloid, chocolate

## Abstract

Caffeine is an alkaloid with a stimulant effect in the body. It can interfere in transmissions based on acetylcholine, epinephrine, norepinephrine, serotonin, dopamine and glutamate. Clinical studies indicate that it can be involved in the slowing of Alzheimer disease pathology and some other effects. The effects are not well understood. In the present work, we focused on the question whether caffeine can inhibit acetylcholinesterase (AChE) and/or, butyrylcholinesterase (BChE), the two enzymes participating in cholinergic neurotransmission. A standard Ellman test with human AChE and BChE was done for altering concentrations of caffeine. The test was supported by an *in silico* examination as well. Donepezil and tacrine were used as standards. In compliance with Dixon’s plot, caffeine was proved to be a non-competitive inhibitor of AChE and BChE. However, inhibition of BChE was quite weak, as the inhibition constant, K_i_, was 13.9 ± 7.4 mol/L. Inhibition of AChE was more relevant, as K_i_ was found to be 175 ± 9 μmol/L. The predicted free energy of binding was −6.7 kcal/mol. The proposed binding orientation of caffeine can interact with Trp86, and it can be stabilize by Tyr337 in comparison to the smaller Ala328 in the case of human BChE; thus, it can explain the lower binding affinity of caffeine for BChE with reference to AChE. The biological relevance of the findings is discussed.

## 1. Introduction

In the body, two structurally close esterases with different functions can be found. While acetylcholinesterase (AChE) (EC 3.1.1.7.) is involved in the termination of neurotransmission, the role of butyrylcholinesterase (BChE) (EC 3.1.1.8.) is not understood. Acetylcholine is a low molecular weight neurotransmitter presented in both the central and peripheral nervous system. It is responsible for signal transmission from nerves to terminal glands and muscles. In the body, nicotinic (nAChR) and muscarinic acetylcholine receptors (mAChR) are present. The receptors are expressed in most tissues, and they can be found on leukocytes, endothelial cells, nerves and others [1–3]. AChE is an enzyme converting acetylcholine into choline and acetate. Neurotransmission is stopped by the AChE effect [4,5].

AChE is a target for many drugs and toxins. Organophosphorus pesticides, carbamate pesticides and nerve agents are examples of toxic compounds inhibiting AChE [6,7]. Huperzine and its derivative, ZT-1, donepezil, galantamine and rivastigmine can be mentioned as drugs for Alzheimer disease inhibiting AChE [8–10]. Compared to drugs for Alzheimer disease penetrating through the blood-brain barrier, drugs for myasthenia gravis, such as pyridostigmine and neostigmine, inhibit AChE in peripheral nerves [11,12]. BChE is not sensitive to all of the AChE inhibitors. Irreversible and pseudo-irreversible inhibitors represented by the aforementioned pesticides, nerve agents, rivastigmine, pyridostigmine and neostigmine have nearly equal affinity to AChE and BChE [4]. BChE does not have a simply defined role in the body. Drugs interacting with the cholinergic system are not focused on BChE for that reason. The enzyme can be found in many tissues, and the expression is not privileged to the closeness of nerves, like in the AChE case. Significant production of BChE can be found in the liver, from where the enzyme is released into the blood system, and it circulates in plasma in a level of 5 mg/mL [13,14].

**Figure 1:**
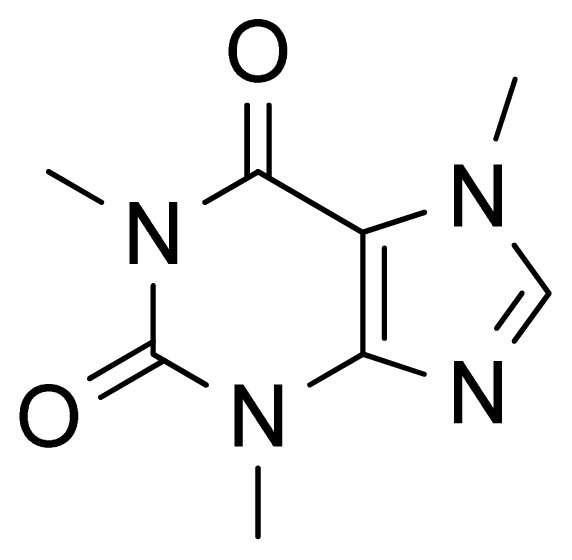
Structure of caffeine.

Caffeine (shown in Figure 1) is a well-known plant alkaloid found in coffee beans from *Coffea arabica*, *C. canephora* and some other *Coffea* plants. It is known to be in tea leaves of *Camellia sinensis* as well. People typically accept caffeine from coffee, tea, energy and cola drinks. Besides the presence in the drinks, caffeine is used as a stimulant supplement and medical stimulant in combination with other compounds [15,16]. The caffeine’s stimulant effect is based on nonselective adenosine receptor antagonism [17]. However, the adenosine receptors are not the only targets of caffeine. It can meet acetylcholine-, epinephrine-, norepinephrine-, serotonin-, dopamine- and glutamate-mediated neurotransmission [18–22]. Phosphodiesterases inhibition and promotion of calcium release from intracellular stores can be attributed to caffeine, as well [23,24]. The implication in the acetylcholine-based neurotransmission is plausibly verified [25]. Caffeine regulatory potency in the body is an object of extensive research, and some of the caffeine related pathways probably remain unrevealed [26]. The present paper describes the search on caffeine potency to modulate the activity of cholinesterases, a crucial part of the cholinergic system. We try to answer the question whether caffeine can act via acetylcholine receptors only or whether it can be involved in the regulation of the neurotransmitter, acetylcholine, via cholinesterases.

## 2. Results and Discussion

Donepezil and tacrine were assayed as standard non-competitive inhibitors of AChE. The inhibitors have no significant affinity to BChE. The inhibition constant, K_i_, for donepezil was assayed to be 23.8 nmol/L. Tacrine was scored to have an inhibition constant, K_i_, equal to 189 nmol/L. The experimental values are in compliance with the literature search, where K_i_ equal to 12.5 nmol/L for donepezil and 105 nmol/L can be found for AChE from rat erythrocytes [27]. The differences between the values subtracted from the literature and the results reported here can be caused by the fact that human AChE was used in our experiment. Small structural alteration between AChE from different organisms can be responsible for the result difference.

**Figure 2:**
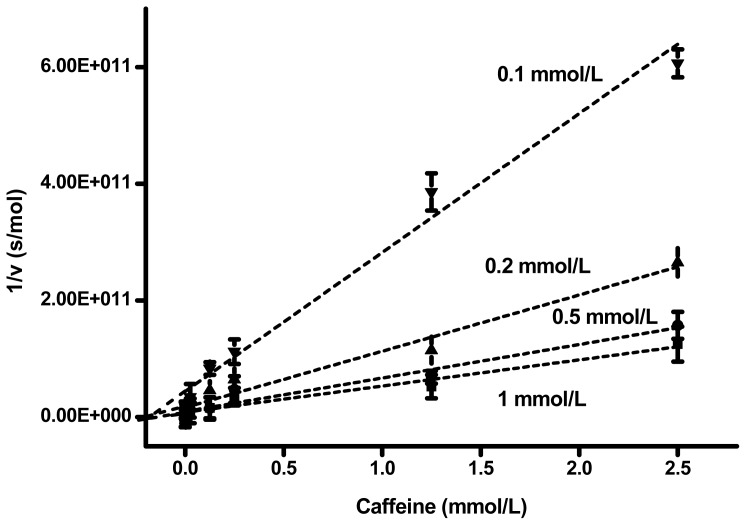
Dixon plot for human acetylcholinesterase (AChE). The concentration of the substrate is indicated beside each line. The data are extrapolated to cross the X-axis. Error bars reflect a standard deviation for *n* = 4.

An assay of caffeine using human AChE is shown in Figure 2. Non-competitive mechanism of inhibition can be easily assumed from the plot. Experimental values for the lines and calculated K_i_ values for each line are depicted in Table 1. The correlation coefficients, R, are quite high, which confirms the precision of the assay. The K_i_ value for caffeine and human AChE was calculated to be (mean ± standard deviation) 175 ± 9 μmol/L. Comparing to AChE, BChE had only minimal sensitivity to inhibition by caffeine. Experimental data for caffeine and human BChE are depicted in Table 2. As the affinity of caffeine to BChE was low, the fitted lines had low slopes, and correlation coefficients were not good for the reason. The K_i_ value for BChE was nearly 80,000 times higher than for AChE: 13.9 ± 7.4 mol/L.

**Figure 3:**
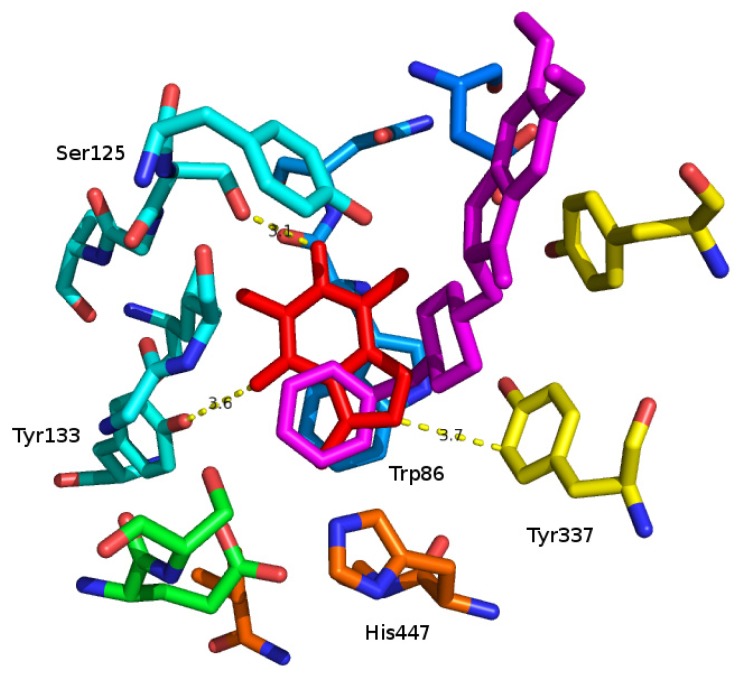
The internal cavity of human AChE is visualized with the predicted binding orientation of caffeine, which is colored by red; the docked orientation of donepezil is colored by violet. Trp86 creating a stacking interaction is situated below the docked orientation of caffeine. Furthermore, there are other important amino acids having an interaction with caffeine (Ser125, Tyr133 and Tyr337). Additionally, His447 creates part of the active site for AChE.

The crystal structure of human AChE with donepezil was considered for docking [28], because it would obtain a more accurate basis for the explanation of the structural feature than the previously available ones. Firstly, donepezil was re-docked to the same binding orientation, as in the crystal structure (the predicted free energy of binding was −12.2 kcal/mol). The phenyl ring was stacked on Trp86 with a π–π interaction. Additionally, galantamine was docked in a similar orientation, as in the crystal structure (pdb code 4ey6 [28]; the predicted free energy of binding is −10.1 kcal/mol). It occupied a whole internal cavity with the active site. Finally, the molecule of caffeine was docked (predicted free energy of binding, −6.7 kcal/mol). The predicted binding orientation of caffeine is stacked toward Trp86 (3.6 Å) with π–π interaction similar to the phenyl cycle of donepezil. Furthermore, there can exist two hydrogen bonds between caffeine and Ser125 (3.1 Å) and a weaker hydrogen bond with Tyr133 (3.6 Å). The binding orientation of caffeine can be stabilized by Tyr337 (3.7 Å) in comparison to human BChE, where it is in the position situated on Ala328, which is not able to extend to the predicted caffeine binding orientation. This can explain the weaker binding activity for BChE in comparison to AChE. The docked orientation of caffeine in the AChE structure is depicted in Figure 3. BChE has very low binding affinity; thus, it was not modeled by docking, as the program is not suitable for such weak interactions.

As seen in the experimental data, caffeine is a selective inhibitor of AChE and not BChE. The fact that caffeine can inhibit AChE can be assumed from some papers [29–31]. The exact mechanism of the inhibition and the detailed comparison of AChE and BChE, however, was not done. Though inhibition of BChE can be found, the inhibition constant is too high to be reached in the body. AChE activity can be affected more easily. It is noteworthy that caffeine is not a highly potent inhibitor of AChE. As seen from the quoted work about tacrine and donepezil, caffeine is approximately a 1000-times weaker inhibitor of AChE than tacrine and a 14,000-times weaker inhibitor than donepezil. On the other hand, caffeine is much less toxic than the mentioned drugs, and it is easier to give a higher dose to the body. The toxic effect of caffeine is assumed when plasmatic concentration reaches 25 mg/L (*i.e.*, 129 μmol/L), and intoxication with caffeine reaching a plasmatic level 85 mg/L (438 μmol/L) is known [32,33]. The ability of caffeine to inhibit AChE was not known at the time of the mentioned case report. However, we can infer that a cholinergic crisis would take place in the intoxicated human, as the reached plasmatic concentration was above the inhibition constant for human AChE. Intake of caffeine in the form of chocolate, coffee, energy drinks or tea probably does not cause significant biological effects based on AChE inhibition. As proved in a study, an amount of caffeine of 100 mg corresponding to one coffee results in a peak concentration of caffeine in plasma of approximately 2.0 mg/L (10.3 μmol/L) for men and 3.6 mg/L (18.5 μmol/L) for women [34]. The quoted plasma concentration is 17-times under the K_i_ in the case of men and 9.5-times under the K_i_ for women. We can estimate that blood AChE is not significantly inhibited when people take one coffee. However, the combination of coffee and energy drink or caffeine tablets can easily reach plasmatic caffeine concentration when the AChE is inhibited.

There was a proven lower incidence of Alzheimer disease in individuals taking coffee regularly [35,36]. This phenomenon is not commonly understood, despite efforts to find key macromolecules in the caffeine pathway [37]. Though some theories were established, none is confirmed on a molecular model. We infer that the inhibition of AChE can be responsible for the effect.

Non-competitive inhibitors blocking anionic sites of AChE are known to be able to improve cognitive functions during Alzheimer disease [5,18,38], and caffeine would act just as the non-competitive inhibitor. Besides the improving cognitive functions, there are hypotheses that the blocking of the peripheral anionic site of AChE could slow down deposition of amyloid plaques [4,5].

The fact that caffeine acts as a selective inhibitor is quite interesting. There is a continuous effort to introduce selective inhibitors in order to ameliorate side effects of drugs [39,40]. Since the etiology of Alzheimer disease is not revealed and many starting mechanisms, including oxidative stress, are currently considered [41–43], the effect of caffeine in clinical studies can be helpful. The findings given in clinical studies [35,36] can be attributed to inhibition of AChE rather than BChE. The second enzyme probably plays another role than AChE in Alzheimer disease development, and both enzymes are suspicious of being involved in the disease’s development [44]. More work on the issue is needed prior to giving a serious explanation.

## 3. Experimental Section

### 3.1. Cholinesterases Activity Assay

Recombinant human BChE and recombinant human AChE were purchased from Sigma-Aldrich (Saint Louis, Missouri, USA). Both enzymes were received as a powder. The BChE had specific activity ≥500 μmol/min/mg of protein and AChE ≥1500 μmol/min/mg. Caffeine, tacrine and donepezil were purchased as analytical standards from Sigma-Aldrich. The other reagents were in a standard purity, and they were received from Sigma-Aldrich, as well.

Cholinesterases inhibition by the tested compounds was assayed using Ellman’s method based on 5,5′-dithiobis-(2-nitrobenzoic) acid as chromogen and, butyrylthiocholine chloride (for BChE) and acetylthiocholine chloride (for AChE) as substrates. The principle and protocol for the assay was published in the quoted papers [45–47]. In the assay, standard PS disposable cuvettes were used. The cuvette was gradually filled with 0.4 mL of 5,5′-dithiobis-(2-nitrobenzoic) acid 0.4 mg/mL, 100 μL of either BChE or AChE solution (1 × 10^−9^ kat for 1 mmol/L substrate and standard ambient temperature and pressure (SATP*)* conditions) in phosphate buffered saline (PBS; composition 137 mmol/L NaCl, 2.7 mmol/L KCl, 10 mmol/L Na_2_HPO_4_, 0.24 mmol/L KH_2_PO_4_, pH 7.4), 100 μL of the tested compound solution in PBS and 300 μL of PBS. The reaction was started by addition of butyrylthiocholine or acetylthiocholine chloride (100 μL). Five minutes after substrate injection to the cuvette, absorbance was measured at 412 nm. Enzyme activity was calculated using the extinction coefficient, ɛ = 14,150 L × mol^−1^ × cm^−1^. The coefficient was taken from the literature describing an experiment where the same assay conditions were used [48].

### 3.2. Molecular Modeling

The first polypeptide chain from the crystal structure of human AChE with donepezil was taken for modeling (pdb code 4EY7) [28]. The water and other molecules were removed, and the molecular program, Sirius (version 1.2, Supercomputer Center, San Diego, CA, USA), was used for preparation of the complex AChE inhibitors. Further structures were modified using the AutoDockTools scripts in order to be docked by AutoDock Vina 1.1.2 [49] with default parameters, where the grid center was situated on the inhibitor, in compliance with the crystal structure, and the grid size was equal to 27 × 18 × 23 Å. The orientation with the lowest free energy of binding was only considered according to the Vina score. The results were visualized with the help of the Pymol software [50].

### 3.3. Statistical Processing of Experimental Data

The experimental data were processed in compliance with Dixon’s method [51,52]. The data were plotted by two ways: as a reciprocal value of velocity against inhibitor concentration and as a substrate concentration divided by reaction velocity against inhibitor concentration, as described by Cornish–Bowden [52]. The inhibition constants, K_i_, were calculated from the plots. Non-competitive standard inhibitors were assayed for their IC_50_. If necessary, the K_i_ value can be derived from the IC_50_ [53,54].

## 4. Conclusions

Caffeine is a simply available drug that has been known for a long time and by many cultures. Despite a lot of work on the identification of caffeine’s effect in the body, some pathways remain undiscovered. In the present work, we proved that caffeine can act as a non-competitive inhibitor of AChE in the body. This finding can be expected in some of the clinically proven effects with no known molecular mechanism. We can emphasize that caffeine can be considered as a potential lead structure in drug design.

## Figures and Tables

**Table 1:** Search on inhibitory mechanism using human AChE.

Substrate (mmol/L)	Slope (s × L/mol ^2^)	Interception (s/mol)	Correlation coefficient	K_i_ (mmol/L)
1	4.49 × 10 ^13^	8.23 × 10 ^9^	0.978	0.183
0.5	5.75 × 10 ^13^	9.43 × 10 ^9^	0.976	0.164
0.2	9.64 × 10 ^13^	1.64 × 10 ^10^	0.981	0.170
0.1	2.38 × 10 ^13^	4.41 × 10 ^10^	0.966	0.185

**Table 2:** Search on inhibitory mechanism using human BChE.

Substrate (mmol/L)	Slope (s × L/mol ^2^)	Interception (s/mol)	Correlation coefficient	K_i_ (mol/L)
5	2.76 × 10 ^8^	6.60 × 10 ^9^	0.277	24.0
1	8.75 × 10 ^8^	5.80 × 10 ^9^	0.609	6.64
0.2	4.62 × 10 ^8^	5.07 × 10 ^9^	0.400	11.0
